# Improved insulin sensitivity and body composition, irrespective of macronutrient intake, after a 12 month intervention in adolescents with pre-diabetes; RESIST a randomised control trial

**DOI:** 10.1186/s12887-014-0289-0

**Published:** 2014-11-25

**Authors:** Sarah P Garnett, Megan Gow, Mandy Ho, Louise A Baur, Manny Noakes, Helen J Woodhead, Carolyn R Broderick, Kerryn Chisholm, Julie Briody, Sukanya De, Katherine Steinbeck, Shubha Srinivasan, Geoffrey R Ambler, Chris T Cowell

**Affiliations:** Institute of Endocrinology and Diabetes, The Children’s Hospital at Westmead, Locked Bag 4001, Westmead, Sydney, NSW 2145 Australia; Kids Research Institute, The Children’s Hospital at Westmead, Locked Bag 4001, Westmead, Sydney, NSW 2145 Australia; The Children’s Hospital at Westmead Clinical School, University of Sydney, Locked Bag 4001, Westmead, Sydney, NSW 2145 Australia; CSIRO Food and Nutritional Sciences, PO Box 10041, Adelaide, BC South Australia 5000 Australia; Department of Paediatrics, Campbelltown Hospital, PO Box 149, Campbelltown, NSW 2560 Australia; Department of Diabetes and Endocrinology, Sydney Children’s Hospital Network, Randwick, Sydney, NSW 2031 Australia; The Children’s Hospital Institute of Sports Medicine, The Children’s Hospital at Westmead, Locked Bag 4001, Westmead, Sydney, NSW 2145 Australia; The School of Medical Sciences, UNSW Medicine, The University of New South Wales, Sydney, NSW 2052 Australia; Nutrition and Dietetics and Weight Management Services, The Children’s Hospital at Westmead, Locked Bag 4001, Westmead, Sydney, NSW 2145 Australia; Department of Nuclear Medicine, The Children’s Hospital at Westmead, Locked Bag 4001, Westmead, Sydney, NSW 2145 Australia; Academic Department of Adolescent Medicine, Sydney Medical School, University of Sydney, Sydney, NSW 2066 Australia

**Keywords:** Insulin sensitivity, Body composition, Macronutrient intake, Adolescents, Pre-diabetes

## Abstract

**Background:**

A higher protein to carbohydrate ratio in the diet may potentiate weight loss, improve body composition and cardiometabolic risk, including glucose homeostasis in adults. The aim of this randomised control trial was to determine the efficacy of two structured lifestyle interventions, differing in dietary macronutrient content, on insulin sensitivity and body composition in adolescents. We hypothesised that a moderate-carbohydrate (40-45% of energy), increased-protein (25-30%) diet would be more effective than a high-carbohydrate diet (55-60%), moderate-protein (15%) diet in improving outcomes in obese, insulin resistant adolescents.

**Methods:**

Obese 10**–**17 year olds with either pre-diabetes and/or clinical features of insulin resistance were recruited at two hospitals in Sydney, Australia. At baseline adolescents were prescribed metformin and randomised to one of two energy restricted diets. The intervention included regular contact with the dietician and a supervised physical activity program. Outcomes included insulin sensitivity index measured by an oral glucose tolerance test and body composition measured by dual-energy x-ray absorptiometry at 12 months.

**Results:**

Of the 111 adolescents recruited, 85 (77%) completed the intervention. BMI expressed as a percentage of the 95th percentile decreased by 6.8% [95% CI: −8.8 to −4.9], ISI increased by 0.2 [95% CI: 0.06 to 0.39] and percent body fat decreased by 2.4% [95% CI: −3.4 to −1.3]. There were no significant differences in outcomes between diet groups at any time.

**Conclusion:**

When treated with metformin and an exercise program, a structured, reduced energy diet, which is either high-carbohydrate or moderate-carbohydrate with increased-protein, can achieve clinically significant improvements in obese adolescents at risk of type 2 diabetes.

**Trial registration:**

Australian New Zealand Clinical Trail Registry ACTRN12608000416392. Registered 25 August 2008.

## Background

There is substantial interest in the effect of the macronutrient composition of the diet on potentiating weight loss and improving cardiometabolic risk [1,2]. Results from several studies indicate that a weight loss diet with increased protein and reduced carbohydrate may increase body fat mass loss, attenuate loss of fat free mass (FFM) and improve lipid profile and glucose homeostasis, compared with a conventional high carbohydrate, low fat diet [3]. It is speculated that protein is superior to carbohydrate in promoting satiety as well as dietary induced thermogenesis, with no unfavourable health implications [4,5]. In addition, high carbohydrate diets may lead to higher post prandial glucose and insulin spikes, placing increased demands on beta cell function and exacerbating insulin sensitivity [6]. However, improved outcomes with an increased protein diet are not consistently reported. A recent systematic review [7] identified three randomised control trials (RCT), conducted in highly controlled environments, two were in residential camps [8,9] and the other in a boarding school [10], comparing increased protein to isoenergetic standard protein diets in obese children; none reported differences in weight loss, cardiometabolic risk, or glycaemic status between diets. However, there is a paucity of trials in free-living overweight or obese adolescents.

We undertook an RCT, known as Researching Effective Strategies to Improve Insulin Sensitivity in Children and Teenagers (RESIST), with the aim of determining the effectiveness of a moderate-carbohydrate, increased protein diet compared to a high carbohydrate diet on insulin sensitivity in adolescents with pre-diabetes and/or clinical features of insulin resistance treated with metformin. We hypothesised that the moderate-carbohydrate, increased-protein diet would be more effective than the high-carbohydrate diet in improving insulin sensitivity, body composition and metabolic profile. The six month results have been previous published and in contrast to our hypothesis, demonstrated no significant differences in weight loss or metabolic profile between dietary groups [11]. The aims of this manuscript are to describe the changes in whole body insulin sensitivity index (ISI), derived from an oral glucose tolerance test (OGTT), and in body composition, measured by dual-energy x-ray absorptiometry (DXA), after 12 months of intervention.

## Methods

This study was a 12 month parallel RCT which took place at The Children’s Hospital at Westmead (CHW) and Campbelltown Hospital, Sydney, Australia. This study was conducted according to the guidelines laid down in the Declaration of Helsinki and was approved by CHW Human Research Ethics Committee (07/CHW/12) and Sydney South West Area Health, Western Zone (08/LPOOL/195). Written informed consent from parents and assent from the adolescents was sought prior to enrolment. The protocol for the study has been published [12]. All participants were treated with metformin and received the same 12 month lifestyle intervention. The only difference between treatment arms was the macronutrient composition of the diet.

The intervention consisted of three phases:

I (0–3 months): Intensive dietary intervention
II (4–6 months): Intensive exercise program plus dietary support
III (7–12 months): Participants were encouraged to continue with their diet/exercise regimens and metformin.

### Study population

Participants were recruited through physician referral [12]. After a patient was assessed and identified as meeting the trial criteria, the patient and parent/carer made contact with study dieticians who explained the study, sent information sheets/consent forms and booked appointments. Treatment allocation (allocation ratio 1:1) occurred centrally by minimisation [13], stratified by sex, pubertal stage and BMI status [14], with the aid of computer software [15] at CHW, by study dieticians.

#### Inclusion criteria

Ten to 17 year old adolescents who were overweight or obese, as defined by the International Obesity TaskForce [14] with either pre type 2 diabetes [16] and/or clinical features of insulin resistance. As previously described clinical features of insulin resistance were defined as a fasting insulin (pmol/L)/glucose (mmol/L) ratio >20 with one or more of the following: acanthosis nigricans, polycystic ovarian syndrome, hypertension, fasting HDL cholesterol <1.03 mmol/L or fasting triglycerides ≥1.7 mmol/L [12].

#### Exclusion criteria

Diabetes, contraindications to metformin, secondary causes of obesity, psychiatric disturbance, significant mental illness, inability to take part in physical activity, weight loss medications or medications known to cause weight gain, and weight >120 kg.

Our target sample size was 108 (54 in each arm). This was based on the primary outcome, change in whole body ISI of 0.8 (SD 1.3), with an 80% chance of detecting a significant increase in ISI at the two sided 5% level and included a 20% dropout rate [12]. A total of 111 entered the study between January 2009 and November 2011, Figure 1.

**Figure 1:**
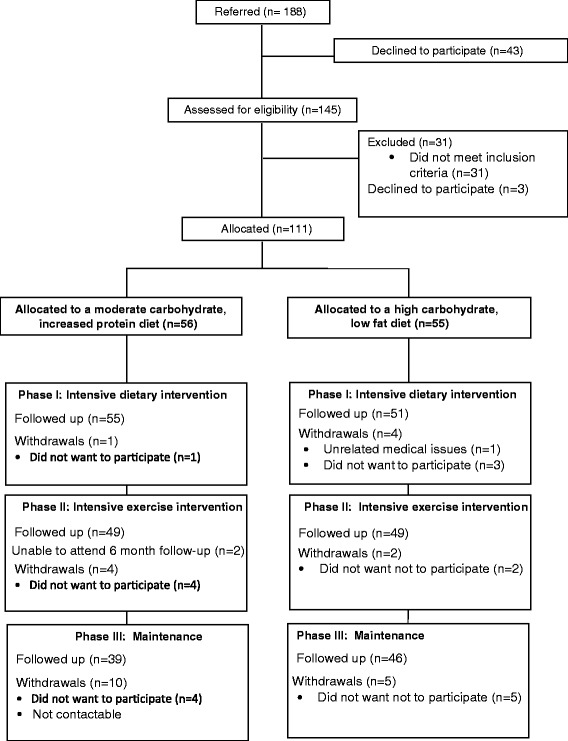
**RESIST participant flow.**

### Interventions

#### Metformin

Consistent with clinical practice at CHW all participants were treated with metformin (Diabex) which was provided at no cost for the duration of the study. The initial dose was 250 mg twice daily. After 2 weeks this was increased to a final dose of 500 mg twice daily.

#### Diet

*Diet 1* was a high-carbohydrate diet, with 55-60% of total energy as carbohydrate (moderate glycaemic load), 30% fat (≤10% saturated fat) and 15% protein. *Diet 2* was a moderate-carbohydrate, increased-protein diet, with 40-45% of total energy as carbohydrate (moderate glycaemic load), 30% fat (≤10% saturated fat), 25-30% protein. Diets were prescriptive at two different energy levels: 6,000 to 7,000 kJ (10 to 14 years) or 7,000 to 8,000 kJ (15 to 17 years). The energy levels were a range for each age group, to enable prescribed energy to individualize, depending upon the energy requirements. Details of the delivery of the intervention have been previously described [12].

#### Exercise

Phase I Standardised physical activity advice, consistent with recommendations for adolescents [17] was delivered by study dieticians.
Phase II Participants received, free of charge, a supervised exercise program, 45–60 minutes, twice/week for 12 weeks in a commercial gym, including Fitness First, or a local park in the geographic area in which they lived. The program was designed to be of moderate-to-vigorous intensity and consisted of circuit training with an age-appropriate mix of resistance and aerobic stations, conducted by qualified fitness trainers, blinded to treatment arm. Participants were also encouraged to exercise at least once a week at home.

#### Medical care

Clinical progress was reviewed by the participant’s primary or study physician, who was blinded to the trial arm of the adolescent and who assessed puberty using Tanner Staging [18], blood pressure, acanthosis nigricans [19] and menstrual history.

### Outcome measures

All measures were undertaken by clinicians blinded to treatment allocation at baseline, three and 12 months*.*

#### Primary outcome

Insulin sensitivity measured at CHW by whole body ISI derived from an OGTT using the following formula:

$$ 10000/\surd \left(\left(\mathrm{Fasting}\ \mathrm{insulin} \times \mathrm{fasting}\ \mathrm{glucose}\right)\times \left(\mathrm{mean}\ 2\mathrm{hr}\ \mathrm{glucose} \times \mathrm{mean}\ 2\mathrm{hr}\ \mathrm{insulin}\right)\right) $$

[20].

#### Secondary outcomes

Change in body composition, anthropometry, acanthosis nigricans, triglycerides, HDL-cholesterol and blood pressure.

### Measurements

Weight and height were measured [21] and BMI z-scores were calculated [22]. BMI was expressed as a percentage of the 95^th^ centile (BMI%95 centile) [23]. Change in BMI z-score is not presented, as >96% of the adolescents had a BMI >97^th^ centile which is beyond the scope of the CDC2000 reference data [24]. Blood pressure was measured using an automated blood pressure monitor (Dinamap 1846SX). Elevated blood pressure was defined as ≥90^th^ centile [25]. An OGTT was performed after an overnight fast [12]. Blood drawn was analysed using standard techniques for lipids, alanine aminotransferase (ALT), gamma-glutamyl transferase (GTT)), and renal function tests (urea, electrolytes, and creatinine). Abnormal triglycerides and HDL-cholesterol were defined as ≥1.7 mmol/L and <1.03 mmol/L, respectively. Elevated hepatic transaminases were defined as ALT and/or GGT ≥1.5 upper limit of 30 U/L [26].

### Body composition

Body composition was measured by DXA (Prodigy, Lunar-GE, Madison,WI USA) equipped with propriety software version13.6. The manufacturer recommended scan mode, as determined by height and weight, and when possible, standard positioning techniques were used. When the adolescent’s width exceeded maximum scan width, the adolescent was “mummy wrapped”, with arms placed in a lateral position. Scans were analysed using manufacturer recommended techniques. Fat mass index (FMI) and FFM index (FFMI) were calculated as fat mass (g)/height (cm)^2^ and FFM (g)/height (cm)^2^, respectively [27].

### Dietary intake

Dietary intake was obtained by 24-hour dietary recalls at 6 weeks and 3, 6 and 12 months. This procedure was conducted by dieticians using a standardised three-pass methodology and food model booklet as previously described [12]. Energy and macronutrient intake were estimated using Foodworks 2009 (version 6.0.2539; Xyris Inc., Brisbane, QLD, Australia).

### Metformin compliance

Adherence was assessed by pill counts by the clinical trials pharmacist at CHW. After three months of intervention, 69 (62%) participants returned pills and after 6 months 50 (45%) participants returned pills. There was no difference between diet groups in adherence at either time period. At three months it was estimated the participants took (median [interquartile range]) 88% [61 to 98] of prescribed metformin and after 6 months participants took 65% [38 to 94]. There was no pill count at 12 months. As previously reported 16 (14%) participants reported side effects to metformin [11].

### Statistical analysis

Data were analysed using PASW statistical software for Windows, version 18 (SPSS Inc). Differences between continuous data were examined using independent sample t-tests for normally distributed data or Mann–Whitney tests for non-parametric data. Chi-squared tests were used to examine differences in categorical data; odds ratios were used to examine the magnitude of the association. Correlations between variables were assessed by Pearson’s correlation coefficients or Spearman’s rho for normally distributed and non-parametric data, respectively. Consistent with an intent-to-treat approach, all available data for participants as originally randomly assigned, were retained. Linear mixed models with an unstructured covariance were used to test for the effects of diet and time (baseline, three, six and 12 months). Non-parametric data were log transformed. Age at baseline and age difference between visits were tested in the modelling but were not statistically and/or clinically significant and hence results have been expressed as unadjusted models. The least significant difference method was used for post-hoc comparisons. The assumptions of modelling were tested and met. Mean changes and differences derived from linear mixed models are presented with 95% CIs. Data that were log transformed are presented as geometric means with 95% CIs.

## Results

Of the 111 adolescents (66 girls) recruited, 85 (77%) completed the 12 month intervention (Figure 1). There was no statistical difference in baseline anthropometry, body composition or clinical parameters between groups (Table 1). The exception was that more participants had pre-diabetes in the moderate-carbohydrate, increased-protein group (n = 11; 19.6%) compared to the high-carbohydrate group (n = 3; 5.5%), P = 0.024. There was also no statistical difference in baseline anthropometry, body composition or clinical parameters between the completers and the drop-outs. Participants who dropped out were more likely to come from a single parent family (odds ratio 4.3 [95% CI: 1.6 to 12.0], P = 0.05). Attrition rate was not statistically significantly different between diet groups (Figure 1). Over the 12 month intervention there was a statistically significant (P < 0.001) decrease in height z-score from 1.27 at baseline to 0.76 at 12 months and the number of children who were pre-pubertal (Tanner stage 1 and 2) decreased from 30.9% to 15.1%. The change in height and pubertal stage was not significantly different between diet groups.

**Table 1:** **Baseline characteristics**

	**Intervention group**				
	**Moderate-carbohydrate, increased-protein diet (n = 56)**		**High-carbohydrate, low-fat diet (n = 55)**		P*
**Age and sex**					
Age years median [range]	13.0	[10.1 to 16.5]	13.2	[10.2 to 17.4]	0.959
Girls n (%)	34	(60.7)	32	(58.2)	0.786^†^
Pubertal status^‡^ n (%)					
Tanner stage 1	7	(12.7)	7	(12.7)	
2	9	(16.4)	11	(20.0)	
3	15	(27.3)	9	(16.4)	
4	15	(27.3)	16	(29.1)	
5	9	(16.4)	12	(21.8)	
**Anthropometry**					
*Mean (SD) unless otherwise indicated*					
Weight kg	90.7	(19.0)	90.7	(21.2)	0.992
Height z-score	1.27	(1.29)	1.30	(1.11)	0.894
Weight z-score	2.73	(0.53)	2.68	(0.57)	0.646
BMI z-score	2.39	(0.25)	2.33	(0.32)	0.253
BMI %95 centile	133	(19)	132	(23)	0.770
Obese^§^ n (%)	55	(98.2)	52	(94.5)	0.300^†^
**Body composition (DXA)** ^|^					
Total body fat (kg)	43.5	(10.0)	42.6	(11.7)	0.683
Total fat %	49.5	(5.5)	48.3	(5.7)	0.249
Total fat free mass (kg)	44.4	(11.3)	45.1	(11.4)	0.740
Fat free mass index	1.66	(0.24)	1.69	(0.29)	0.638

*P independent sample t-tests unless otherwise indicated.

^†^Chi-squared test.

^‡^One girl in the moderate carbohydrate, increase protein diet group had missing data.

^§^Obesity defined by International Obesity Taskforce [14].

^|^DXA: Dual-energy x-ray absorptiometry.

### Effects of intervention on insulin sensitivity index

**Figure 2:**
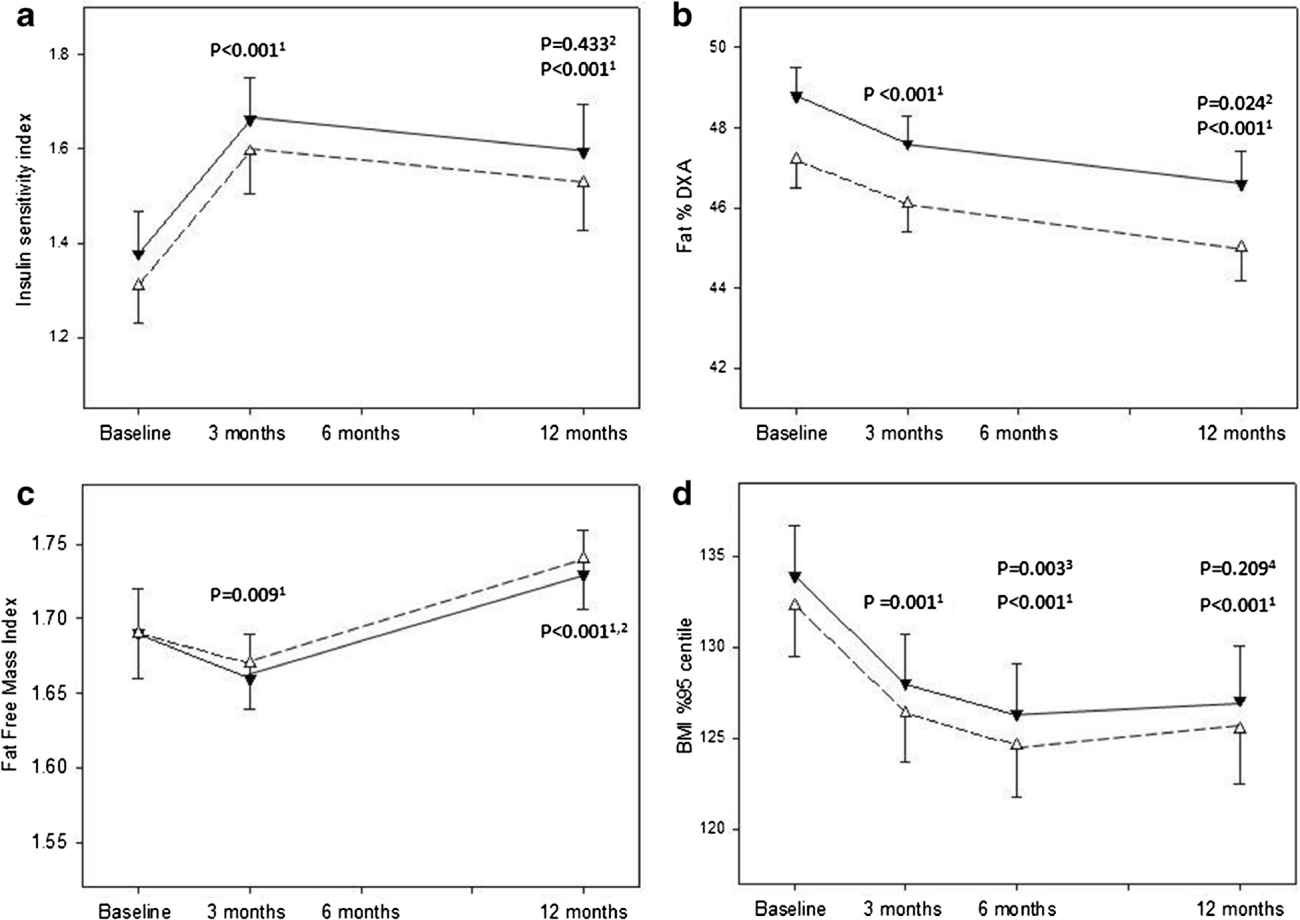
**Glycemic status and body composition measures by dietary group over the 12 month intervention.** Estimated marginal means (SE) are presented from linear mixed models for the moderate-carbohydrate, increased-protein diet group (▼) and the high-carbohydrate diet group (△). **a**: Insulin sensitivity index. ^1^Significance between baseline and 3 months and 12 months as indicated. ^2^Significance between 3 and 12 months. **b**: Total body fat percent measured by dual energy x-ray absorptiometry (Fat % DXA). ^1^Significance between baseline and 3 months and 12 months as indicated. ^2^Significance between 3 and 12 months. **c**: Fat free mass index. ^1^Significance between baseline and 3 months and 12 months as indicated. ^2^Significance between 3 and 12 months. **d**: BMI%95th centile. ^1^Significance between baseline and 3 months, 6 months and 12 months as indicated. ^3^Significance between 3 and 6 months. ^4^Significance between 6 and 12 months.

ISI increased between baseline and three months, which remained significantly different from baseline at 12 months; estimated mean difference 0.23 [0.06 to 0.39], P = 0.009; Figure 2a. The magnitude of change was similar for girls and boys, although boys commenced the trial with a lower median ISI (1.2 [range 0.3 to 3.0]) compared to girls (1.4 [0.3 to 3.4]), P = 0.04. Adjusting for puberty and/or age did not alter the outcome. There was no significant difference in ISI between diet groups at any time point.

### Effects of intervention on total body fat

Total body fat% decreased over the 12 month intervention (P < 0.001, Figure 2b). There was a significant sex and pubertal interaction in the modelling. After adjusting for pubertal stage, the magnitude of change was similar for boys and girls, however, boys commenced the trial with a lower total body fat percentage (46.3% ± 5.3) compared to girls (50.7% ±5 .3), P < 0.001. There was no significant difference in total body fat% between diet groups at any time point. Similarly the FMI significantly (P = 0.009) decreased from 16.2 ± 0.4 (estimated marginal mean ± SE) at baseline to 15.6 ± 0.5 at 12 months and there was no significant difference (P = 0.421) in FMI between diet groups at any time point.

### Effects of intervention on fat free mass index

After three months of intervention there was a significant decrease in FFMI, followed by a significant increase between three and 12 months (Figure 2c). Analysis stratified by sex indicated that girls, but not boys, had a significant decrease; estimated mean difference (0.03 [95% CI: 0.01 to 0.05], P = 0.005) in the FFMI during the first three months, which increased (0.03 [0.001 to 0.06], P = 0.014) to baseline levels at 12 month. Boys’ FFMI did not change over the first three months, but FFMI was higher at 12 months compared to both baseline (0.09 [0.06 to 0.12], P < 0.001]) and three months (0.11 [0.06 to 0.15], P < 0.001). Adjusting for puberty and/or age did not alter the outcome. There was no significant difference between diet groups at any time point.

### Effects of intervention on BMI%95th centile

BMI%95th centile decreased between baseline and 12 months (P < 0.001, Figure 2d). The decrease in BMI%95th centile occurred between baseline and six months and there was no significant change over the following six months. The magnitude of change (estimated mean difference 6.8% [95% CI: 4.9 to 8.8]) over the 12 months was similar for boys and girls, although boys commenced the trial with a higher mean (±SD) BMI%95^th^ centile compared to girls; 136.8 ± 21.1 and 129.3 ± 20.6, P = 0.020, respectively. There was no significant difference between diet groups at any time point.

### Effects of intervention on lipids and blood pressure

Between baseline and 12 months there was a significant increase in HDL-cholesterol and a significant decrease in diastolic blood pressure (Table 2). There were no significant differences between baseline and 12 month measures of LDL-cholesterol , triglycerides or systolic blood pressure, nor was there any significant difference in lipids or blood pressure between diet groups at any time point. Sex was not a significant predictor of change in HDL-cholesterol, LDL-cholesterol or triglycerides. However, a sex difference in blood pressure was observed. The magnitude of change in both SBP z-score and DBP z-score over the 12 months was similar for boys and girls, although boys commenced the trial with a higher blood pressure. At baseline the SBP z-score (estimated marginal mean (SE)) for boys and girls was 1.10 (0.14) and 0.49 (0.16), respectively and DBP was 1.13 (0.09) and 0.75 (0.11), respectively.

**Table 2:** **Lipids and blood pressure at baseline and 12 months**

	**Baseline**		**After 12 months intervention**		**P** *
Triglycerides mmol/L^†^	1.1	[1.0-1.2]	1.0	[0.9-1.1]	0.209
HDL-C mmol/L	1.05	(0.02)	1.12	(0.03)	<0.001
LDL-C mol/L	2.77	(0.08)	2.75	(0.08)	0.456
SBP z-score	0.79	(0.12)	0.65	(0.12)	0.270
DBP z-score	0.94	(0.08)	0.74	(0.10)	0.047

*Pairwise comparison with baseline.

^†^Geometric mean [95% CI].

Estimated marginal means (SE) are presented from linear mixed models, unless otherwise indicated.

### Dietary adherence

The geometric mean [95% CI] for the reported energy intake over the 12 month intervention was 5.97 [5.94 to 6.37] MJ per day for adolescents randomised to the moderate-carbohydrate, increased-protein diet and 6.41 [6.00 to 6.85] MJ per day for adolescents randomised to the high-carbohydrate diet. The difference was not significant (P = 0.126), nor did the reported energy intake differ over time (P = 0.935). Protein % energy was significantly (P = 0.027) higher in the moderate-carbohydrate increased-protein group compared to the high-carbohydrate group, 20.3% [19.3 to 21.2] and 18.8% [17.8 to 19.2], respectively, and this did not differ over time (P = 0.081). There was no statistical difference in reported fat % energy (both groups 30.4% [28.5 to 32.2], P = 0.710) or carbohydrate % energy (46.5% [44.7 to 48.1] for the increased-protein group and 48.2% [46.4 to 49.9] for the high-carbohydrate group, P = 0.155). Fat % energy intake did not change significantly over time (P = 0.191). In contrast, reported carbohydrate % energy was significantly higher in both groups at six months compared to six weeks, three months and 12 months, P = 0.003.

### Clinical outcomes in adolescents who completed the intervention

There was no statistical difference in clinical outcomes between diet groups at any time point; data has been pooled for this analysis, Table 3.

**Table 3:** **Clinical presentation at baseline, 3, 6 and 12 months**

	**Baseline**		**3 months**		**6 months**		**12 months**	
	**n = 111**		**n = 106**		**n = 100**		**n = 85**	
Acanthosis nigricans present	94	(84.7)	88	(83.8)*	79	(85.9)^†^	61	(76.3)^§^
Pre-diabetes	14	(12.6)	19	(17.9)			10	(12.1)^†,^**
Dyslipidaemia	61	(55.0)*	57	(54.3)*	51	(53.7)^|^	32	(28.8)^†^
(HDL-C <1.03 mmol/L and/or triglycerides ≥1.7 mmol/L)								
SBP and/or DBP ≥90 percentile	49	(44.1)	41	(38.7)	30	(30.6)^†^	37	(43.5)
Elevated liver enzymes (ALT and/or GGT ≥1.5 upper limit of 30 U/L)	22	(20.0)^†^	21	(20.4)^‡^	17	(17.3)^†^	14	(12.6)^‡^
Microalbuminuria	9	(8.5)^§^	9	(8.9)^§^	9	(9.5)^§^	9^§^	(8.1)^§^
(Urine/albumin/creatinine, Girls: 3.5 to 25 mg/mmol, Boys: 2.5 to 25 mg/mmol)								

*1 missing value, ^†^2 missing values, ^‡^3 missing values, ^§^5 missing values, ^**|**^ 8 missing values **including one adolescent diagnosed with type 2 diabetes.

Values are n (%).

#### BMI %95th centile

Of the 85(82 obese at baseline) who completed the study, 67 (78.8%) decreased BMI %95th centile and 18 increased BMI %95th centile. Two participants completed the 12 month intervention with a weight within the normal range, and 12 were classified as overweight. Baseline sex, age, puberty, weight, or fasting insulin were not significantly associated with change in BMI %95th centile. However, participants who entered the trial with a higher ISI, lost less weight (BMI %95th centile), rho −0.26, P = 0.018.

#### Pre-diabetes

Eighty-three participants had glycaemic status measured at baseline and 12 months. Eight (9.6%) of the 83 participants had pre-diabetes at baseline (three impaired fasting glucose (IFG), four impaired glucose tolerance (IGT), one with both IFG and IGT), but only 2 had pre-diabetes at 12 months. The six participants who improved glycaemic status lost significantly more weight. The mean BMI %95th centile decreased by −21.3 [95% CI:-34.3 to −8.2]), total fat % decreased by −8.7 [−22.3 to 1.7] and ISI increased by 1.0 [0.02 to 3.6]. An additional 8 participants developed pre-diabetes over the 12 months and one, an 11 year old boy, gained 16 kg and developed type 2 diabetes.

#### Acanthosis nigricans

Eighty participants were assessed for acanthosis nigricans at baseline and 12 months, of which 66 (82.5%) entered the trial with acanthosis nigricans. After the 12 month intervention acanthosis nigricans resolved in seven participants and developed in two. Those who had resolution lost more total body fat % compared to those who did not, mean difference in total body fat % 4.7 [95% CI:1.6 to 7.8], but there was no significant difference in ISI.

#### Dyslipidemia

Eighty-two participants had blood lipids measured at baseline and 12 months, of which 46 (56.1%) entered the trial with dyslipidaemia. After 12 months of intervention, dyslipidaemia resolved in 19 (23.2%) participants and developed in 5 (6.1%). There were no statistical differences in change in BMI %95^th^ centile, total body fat% or ISI between those who did or did not have resolution.

#### Blood pressure

All participants who completed the interventions had blood pressure measured at baseline and 12 months, of which 49 (44.1%) entered the trial with elevated blood pressure. After 12 months of intervention, blood pressure decreased to normal levels in 12 (14.1%) participants and increased in 12 (14.1%). There was no statistical difference in the number of participants with elevated blood pressure at any time point (P = 0.183). There were no statistical differences in change in BMI %95th centile, total body fat% or ISI between those who did or did not have improved blood pressure.

## Discussion

Overall, results from this study indicate that a 12 month lifestyle intervention combined with metformin therapy in overweight and obese adolescents at risk of developing type 2 diabetes was effective in achieving moderate improvement in body composition and BMI. Pre-diabetes and clinical features including acanthosis nigricans, also improved, particularly in adolescents who lost weight (BMI % 95^th^ centile) and/or total body fat %. ISI also increased significantly; however, the magnitude of difference between baseline and 12 months was small and may not be clinically significant. In contrast to our hypothesis, that adolescents randomised to a moderate-carbohydrate, increased-protein diet would have better outcomes compared to the high-carbohydrate diet, the diets had no differential effect on any outcome measure, at any time point. These results are consistent with three other RCTs in overweight and obese adolescents that investigated the effect of varying protein content compared with control diets on weight loss in residential settings [7] but in contrast to those from a recent systematic review of RCTs in overweight and obese adults [3]. To our knowledge, there is only one study, the Diogenes study, which has shown a beneficial effect of increasing the protein in the diet, particularly when coupled with a low glycaemic diet, on both body fat and cardiometabolic markers in children [28,29]. There are a number of reasons why the results may differ from our study and others, including study design; in the Diogenes study *families* were randomised, not children and the focus was children at risk of obesity and weight maintenance, not obese children and weight loss.

The lack of effect between diets in our study may be due to poor compliance. Many participants had difficulty in achieving the macronutrient goals of the prescribed diet and the mean difference in protein intake was marginal (<2% of energy). However, the results remained unchanged in post-hoc analysis of those who were able to meet the targets. Our study was undertaken in a real-life setting and it is not evident if lack of compliance is a consequence of inadequate protein targets or a consequence of readily available high carbohydrate snack foods.

The specific effect of metformin therapy on outcome measures in our study is not clear. The beneficial effects of metformin therapy combined with lifestyle interventions in adolescents with clinical features of insulin resistance are well documented [30,31]. However, results from the largest randomised, placebo controlled trial of metformin alone, on weight and metabolic markers in 150 obese adolescents with hyperinsulinemia and/or pre-diabetes, indicated no significant change in ISI after three and six months of metformin therapy [32]. There are no RCTs which have compared lifestyle interventions to metformin therapy alone in adolescents. We speculate that it is the *combined* effect of metformin therapy and lifestyle intervention which resulted in weight loss and improved glycaemic status in our study. It should also be noted that the improvement in ISI occurred during puberty, a time when insulin sensitivity is expected to decrease irrespective of body composition [33].

The magnitude of total fat % loss after the 12 month intervention was small (−2.4%), although similar to other studies examining the impact of dietary and exercise interventions in obese adolescents and children [30]. However, we may have expected many of the participants to be increasing body fat as part of normal growth and development during puberty [24]; the number of children who were pre-pubertal (Tanner stage 1 and 2) decreased from 30.9% to 15.1% over the 12 months. Loss of total fat% was not associated with resolution of dyslipidemia or lowering of blood pressure; previous studies have reported mixed results [34].

Limitations of the study including the use of ISI to measure glycaemic status, proxy measures of dietary compliance (24 hour recalls) and non-blinding of participants and dieticians. Lack of baseline dietary intake meant that we were unable to determine whether intake was altered by either dietary intervention. However, both groups lost similar amounts of weight, indicating that the energy deficit is likely to be similar in both diet groups. Another limitation was that metformin adherence was not measured at 12 months, hampering our interpretation of the effect of lifestyle compared to metformin therapy.

A strength of the study was the retention rate. After the six month intensive lifestyle intervention, including regular dietary counselling, food hampers to support the prescribed diet, a three month supervised physical activity program and email support, we retained 88% of those recruited. After 12 months, the last six months being a maintenance phase with regular, but limited contact with health professionals, we retained 77%. The challenges of recruitment and retention of adolescents have been previously described [35]. Most (77%) adolescents that dropped out reported lack of interest, highlighting that one program does not suit everyone and alternative approaches to managing adolescents with insulin resistance/pre-diabetes are required. Food preferences are personal; dietary modification may need to be individualised.

## Conclusion

Reduced energy intake, combined with physical activity and assisted by metformin, is likely to be the mainstay for improving insulin sensitivity in this large RCT, completed in a challenging developmental stage. We were unable to demonstrate that the two study diets had differential effects on ISI, body composition or BMI, at any time point. This finding and the improvement in acanthosis nigricans, as a clinical indicator of insulin resistance suggest that a prescribed reduced energy diet is the important intervention message rather than diet composition for overweight and obese adolescents at risk of type 2 diabetes.

## Abbreviations

FFM: Fat free mass
RCT: Randomised control trial
RESIST: Researching Effective Strategies to Improve Insulin Sensitivity in Children and Teenagers
OGTT: Oral glucose tolerance test
BMI%95 centile: BMI expressed as a percentage of the 95th centile
ISI: Insulin sensitivity index
DXA: Dual energy x-ray absorptiometry
CHW: The Children’s Hospital at Westmead
ALT: Alanine aminotransferase
GTT: Gamma-glutamyl transferase
FMI: Fat mass index
FFMI: Fat free mass index
